# Human adipose-derived stem cells promote seawater-immersed wound healing via proangiogenic effects

**DOI:** 10.18632/aging.202773

**Published:** 2021-03-26

**Authors:** Jiachao Xiong, Hong Qiang, Ting Li, Jiayi Zhao, Ziyu Wang, Fei Li, Jianwen Xu

**Affiliations:** 1 Department of Plastic Surgery, Shanghai East Hospital, Tongji University School of Medicine, Shanghai 200120, China; 2 Department of Nursing, Shanghai Yangpu Shidong Hospital, Shanghai 200438, China; 3 Department of General Practice, Changhai Hospital, Naval Military Medical University, Shanghai 200433, China; 4 Department of Neurology, Shanghai Yangpu Shidong Hospital, Shanghai 200438, China; 5 Department of Wound Stoma Clinic, Shanghai Yangpu Shidong Hospital, Shanghai 200438, China

**Keywords:** human adipose-derived stem cell, seawater, wound healing, angiogenesis

## Abstract

Seawater immersion can increase the damage to skin wounds and produce chronic wounds, and the application of human adipose-derived stem cells can significantly promote healing. However, the mechanism underlying angiogenesis is currently unclear. In this study, we investigated the vascularization effect of human adipose-derived stem cells on the repair of seawater-treated skin wounds and explored the underlying mechanisms using bioinformatics. The results showed that human adipose-derived stem cells differentiated into vascular endothelial cells and promoted seawater-immersed wound vascularization by promoting vascular endothelial cell proliferation and migration. The differentially expressed genes between human adipose-derived stem cells and fibroblasts were identified and analyzed (including via gene ontology and Kyoto Encyclopedia of Genes and Genomes pathway enrichment, protein–protein interaction network, and correlation analyses). The genes may promote wound healing by regulating the mechanisms of extracellular matrix remodeling, programmed cell death, inflammation, and vascularization. In conclusion, this study provides novel insights into the use of human adipose-derived stem cells in the regeneration of seawater-immersed skin wounds and chronic wounds.

## INTRODUCTION

Chronic wounds have become a global concern and can be caused by multiple factors, such as diabetes, obesity, and persistent infection, which are characterized by anatomical and functional integrity impairment and not closing within 30 days [1]. Long-term nonhealing wounds increase patient hospitalization time and expenses and eventually lead to serious consequences, such as amputation or death [2]. Seawater is characterized by hypertonic alkalinity because its main chemical components are inorganic salts, such as NaCl, KCl, CaCl_2_, MgCl_2_, and MgSO_4_, which also contain a large number of microorganisms, especially those rich in Gram-negative bacteria [3]. Skin wounds immersed in seawater for a long period of time develop an enlarged area of tissue necrosis and enhanced inflammatory response, which makes wound closure difficult. Therefore, seawater immersion has become a common cause of chronic wounds in people living in coastal areas and participating in ocean navigation.

Wound healing includes multiple stages of hemostasis, inflammation, proliferation, and remodeling. The obstruction of any stage can postpone the healing process and hinder chronic wounds, while vascularization is a crucial factor for the successful closure of chronic wounds. Stem cell regeneration therapy, a new direction of damage repair, has made great progress in the treatment of chronic wounds. Adipose-derived stem cells (ADSCs) derived from adipose tissue are a type of mesenchymal stem cell. ADSCs can chemoattract to the damaged site and differentiate into keratinocytes, fibroblasts, vascular endothelial cells, and other components of skin accessory tissues to be involved in wound repair [4–6]. At the same time, ADSCs secrete various growth factors, such as basic fibroblast growth factor, vascular endothelial growth factor (VEGF), hepatocyte growth factor, and platelet-derived growth factor, to regulate the inflammatory microenvironment of the wound and accelerate wound local micro-vascularization [7, 8]. However, the mechanism of angiogenesis promotion remains unclear.

Our previous study confirmed that seawater immersion significantly delays wound healing, and that human ADSCs (hADSCs) can differentiate into skin stem cells and increase the proliferation and migration of autologous skin stem cells to promote the repair of seawater immersion wounds [9]. However, the effect and mechanism of vascularization have not yet been explored. Therefore, we observed the proangiogenic effects of hADSCs used in seawater immersion wounds *in vivo* and *in vitro*. Bioinformatics technology was used to identify differentially expressed genes (DEGs) in hADSCs and to screen for their main biological functions and action pathways. Meanwhile, the correlation between DEGs and gene markers of angiogenesis, necroptosis, pyroptosis, ferroptosis, and anti-inflammatory activity was explored. The results indicated that hADSCs, via their interaction with vascular endothelial cells, increase the proliferation and migration ability of vascular endothelial cells, which promotes the vascularization of chronic wounds.

## RESULTS

### The angiogenic effect of hADSCs in seawater-immersed wounds

**Figure 1:**
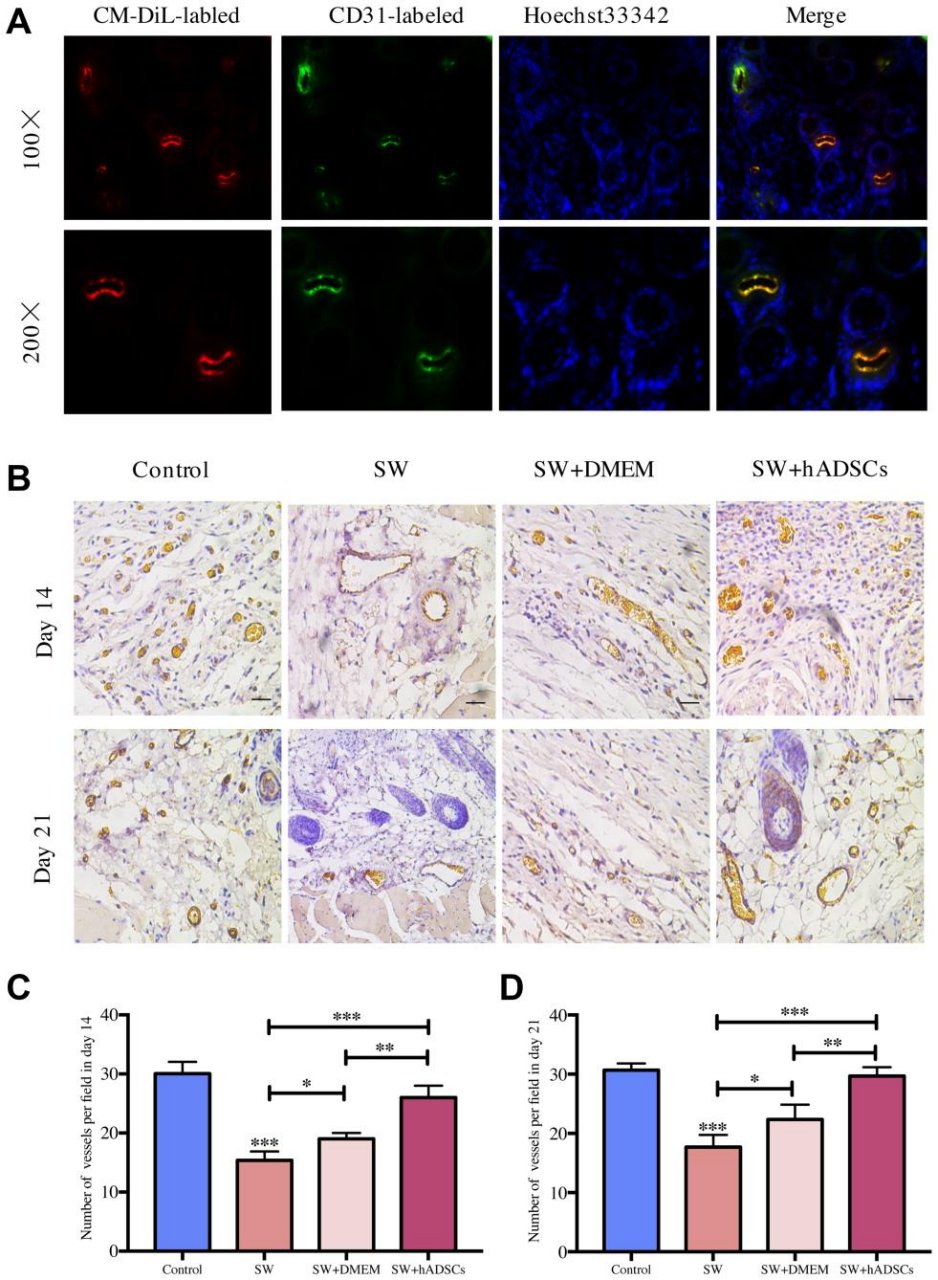
**Microscopic evaluation of wound proangiogenic repair after SW and hADSC treatments.** (**A**) the frozen section immunofluorescence showed that hADSCs (CM-DIL, red) differentiated into vascular endothelial cells (CD31, green) to promote wound healing. (**B–D**) the number of vessels per field in each group at day 14 and day 21. Scale bars indicate 100 μm. ^*^P < 0:05, ^**^P < 0:01, and ^***^P < 0:001.

The level of wound vascularization is an important indicator for evaluating the effects of wound healing. We previously reported that seawater immersion significantly delays wound healing and the treatment of hADSCs can promote wound healing. Therefore, we further analyzed the effects of seawater and hADSCs on wound angiogenesis. Fluorescence staining of frozen sections (Figure 1A) showed that hADSCs could differentiate into vascular endothelial cells in damaged wounds to promote wound healing. Subsequently, CD31 immunohistochemical staining was performed on the wound sections to evaluate repaired wound vascularization. The results (Figure 1B–1D) showed that the numbers of new blood vessels in the control and the SW + hADSC groups were significantly higher than those in the SW group on days 14 and 21 (*P* < 0.001). These results suggest that seawater immersion can significantly delay the neovascularization of wounds, while hADSCs reduce the damage caused by seawater immersion and promote wound vascularization.

### Identification of DEGs in hADSCs

**Figure 2:**
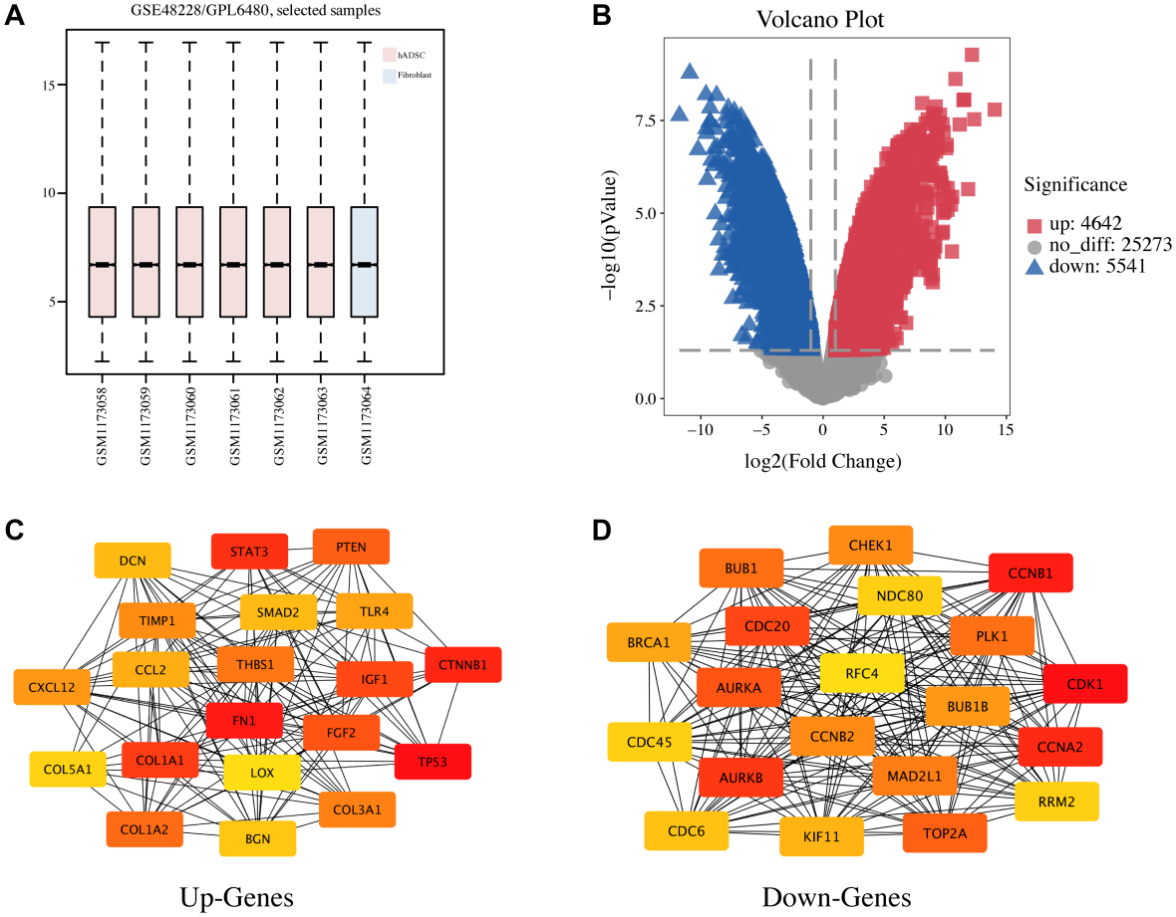
**Identification of DEGs and the PPI network analysis in hADSC.** (**A**) the GSE48228 microarray dataset obtained from the GEO database after normalization. (**B**) volcano plot of the DEGs. (**C**) the mapped networks for the top 20 hub upregulated genes. (**D**) the mapped networks for the top 20 hub downregulated genes.

To explore the potential vascularization mechanism of hADSCs to promote the healing of seawater immersion wounds, a gene expression microarray dataset (GSE48228) was downloaded. The data were normalized using the limma R package, and the mRNA expression levels of the two groups were compared (Figure 2A). The screening criteria were set to a *p* value of < 0.05, and |log FC| > 2, and the results showed 4,642 upregulated and 5,541 downregulated genes, and volcano maps were used to show the distribution of all DEGs (Figure 2B). The top 10 DEGs were selected and are listed in Table 1. DEGs were sorted according to the *p* value; DCN, XIST, and COX7A1 were the top three upregulated DEGs, and LCN2, CALB1, and COX7B2 were the top three downregulated DEGs.

**Table 1:** Top ten up-regulated and down-regulated DEGs between hADSC and fibroblast.

**Gene Name**	**adj. *P*-Value**	***P*-Value**	***t***	**B**	**logFC**	**Regulated**
DCN	1.88E–05	5.30E–10	34.17	10.41	12.18	Up-regulated
XIST	2.83E–05	2.39E–09	28.29	9.84	10.83	Up-regulated
COX7A1	4.49E–05	8.62E–09	24.09	9.25	11.55	Up-regulated
COL3A1	4.49E–05	8.94E–09	23.98	9.23	11.49	Up-regulated
CTNNA1	4.49E–05	1.07E–08	23.44	9.14	8.10	Up-regulated
CDH13	4.49E–05	1.33E–08	22.81	9.02	9.22	Up-regulated
COL1A2	4.49E–05	1.61E–08	22.27	8.92	14.04	Up-regulated
CRLF1	4.49E–05	2.03E–08	21.62	8.79	8.99	Up-regulated
HSPB2	4.49E–05	2.20E–08	21.41	8.74	9.57	Up-regulated
CTNNA1	4.49E–05	2.61E–08	20.95	8.64	9.05	Up-regulated
LCN2	2.83E–05	1.67E–09	–29.60	9.99	–10.91	Down-regulated
CALB1	4.49E–05	6.34E–09	–25.04	9.40	–9.58	Down-regulated
COX7B2	4.49E–05	6.68E–09	–24.88	9.38	–8.71	Down-regulated
FOXA1	4.49E–05	1.44E–08	–22.59	8.98	–9.23	Down-regulated
No Name	4.49E–05	1.48E–08	–22.50	8.96	–7.76	Down-regulated
ADGRF1	4.49E–05	1.79E–08	–21.97	8.86	–7.27	Down-regulated
MANEAL	4.49E–05	2.30E–08	–21.29	8.72	–7.89	Down-regulated
MAL2	4.49E–05	2.33E–08	–21.26	8.71	–11.75	Down-regulated
LINC01351	4.49E–05	2.52E–08	–21.05	8.66	–6.71	Down-regulated
CELSR1	4.49E–05	2.92E–08	–20.66	8.58	–7.15	Down-regulated

### Protein–protein interaction (PPI) network and enrichment analysis of the DEGs

PPI network and enrichment analyses are important for understanding the biological functions and signaling pathways of genes and the functional connections between proteins. The STRING database was used to perform PPI network analysis on the top 2,000 most upregulated and downregulated DEGs. Then, the degree algorithm was used to obtain the hub genes with the highest degree of the first 20 nodes (Table 2) and these were visualized using Cytoscape software (Figure 2C, 2D). TP53, FN1, CTNNB1, STAT3, and COL1A1 as well as CDK1, CCNB1, CCNA2, AURKB, and CDC20 were the top five hub genes for the upregulated and downregulated DEGs, respectively.

**Table 2:** Hub genes in the PPI networks.

**Up-regulated Genes**		**Down-regulated Genes**	
**Gene**	**Degree**	**Gene**	**Degree**
TP53	200	CDK1	271
FN1	176	CCNB1	232
CTNNB1	117	CCNA2	223
STAT3	115	AURKB	218
COL1A1	108	CDC20	215
IGF1	107	AURKA	213
FGF2	104	TOP2A	211
PTEN	99	PLK1	210
COL1A2	93	BUB1	210
THBS1	91	MAD2L1	207
COL3A1	90	CCNB2	200
TIMP1	80	CHEK1	200
CXCL12	78	BUB1B	198
TLR4	76	BRCA1	196
CCL2	75	KIF11	193
SMAD2	74	CDC6	190
DCN	74	NDC80	189
BGN	73	CDC45	189
COL5A1	72	RRM2	189
LOX	70	RFC4	187
TP53	200	CDK1	271
FN1	176	CCNB1	232
CTNNB1	117	CCNA2	223
STAT3	115	AURKB	218
COL1A1	108	CDC20	215

**Figure 3:**
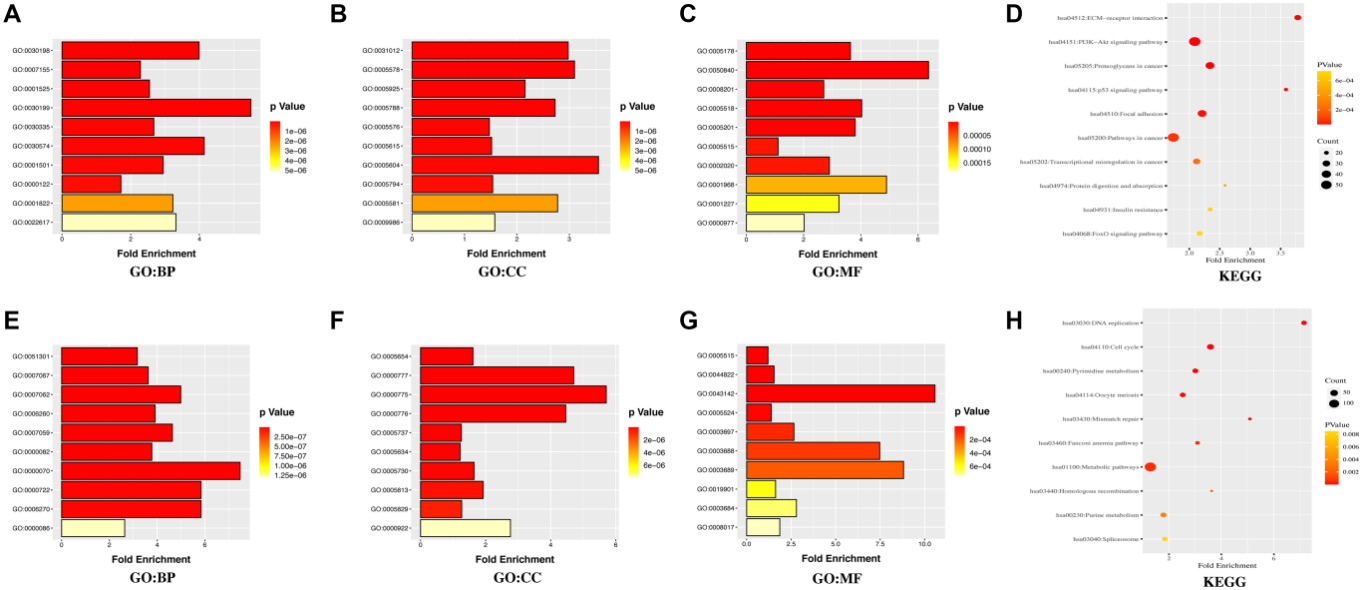
**GO annotation and KEGG pathway enrichment analysis of the DEGs through the DAVID database.** (**A, E**) the category of “biological process” of upregulated DEGs and downregulated DEGs, respectively. (**B, F**) the category of “cellular component” of upregulated DEGs and downregulated DEGs, respectively. (**C, G**) the category of “molecular function” of the predicted TG of upregulated DEGs and downregulated DEGs, respectively. (**D, H**) the category of “KEGG” of upregulated DEGs and downregulated DEGs, respectively.

Furthermore, the database for annotation, visualization, and integrated discovery (DAVID) was used for gene ontology (GO) annotation and the Kyoto Encyclopedia of Genes and Genomes (KEGG) pathway analysis to further reveal the enrichment status of DEGs in the molecular functions (MFs), biological processes (BPs), cell components (CCs), and pathways. Regarding BPs (Figure 3A, 3E), the upregulated DEGs were mainly involved in GO:0030198 (extracellular matrix (ECM) organization), GO:0007155 (cell adhesion), and GO:0001525 (angiogenesis), while the downregulated DEGs were mainly involved in GO:0051301 (cell division), GO:0007067 (mitotic nuclear division), and GO:0007062 (sister chromatid cohesion). Regarding CCs (Figure 3B, 3F), the majority of upregulated DEGs were components of GO:0031012 (ECM), GO:0005578 (proteinaceous ECM), and GO:0005925 (focal adhesion), whereas the downregulated DEGs were mainly components of GO:0005654 (nucleoplasm), GO:0000777 (condensed chromosome kinetochore), and GO:0000775 (chromosome, centromeric region). For the MFs (Figure 3C, 3G), the upregulated DEGs were mainly involved in GO:0005178 (integrin binding), GO:0050840 (ECM binding), and GO:0008201 (heparin binding), whereas the downregulated DEGs were mainly involved in GO:0005515 (protein binding), GO:0044822 (poly(A) RNA binding), and GO:0043142 (single-stranded DNA-dependent ATPase activity). KEGG pathway analysis was used to define the pathways and functions of the DEGs (Figure 3D, 3H). Most upregulated DEGs were significantly enriched in hsa04512 (ECM-receptor interaction), hsa04151 (phosphoinositide 3 kinase (PI3K)-Akt signaling pathway), and hsa05205 (proteoglycans in cancer), whereas the downregulated DEGs were mainly enriched in hsa03030 (DNA replication), hsa04110 (cell cycle), and hsa00240 (pyrimidine metabolism).

### Relationships between DEGs and angiogenesis, necroptosis, pyroptosis, ferroptosis, and anti-inflammatory activities

**Figure 4:**
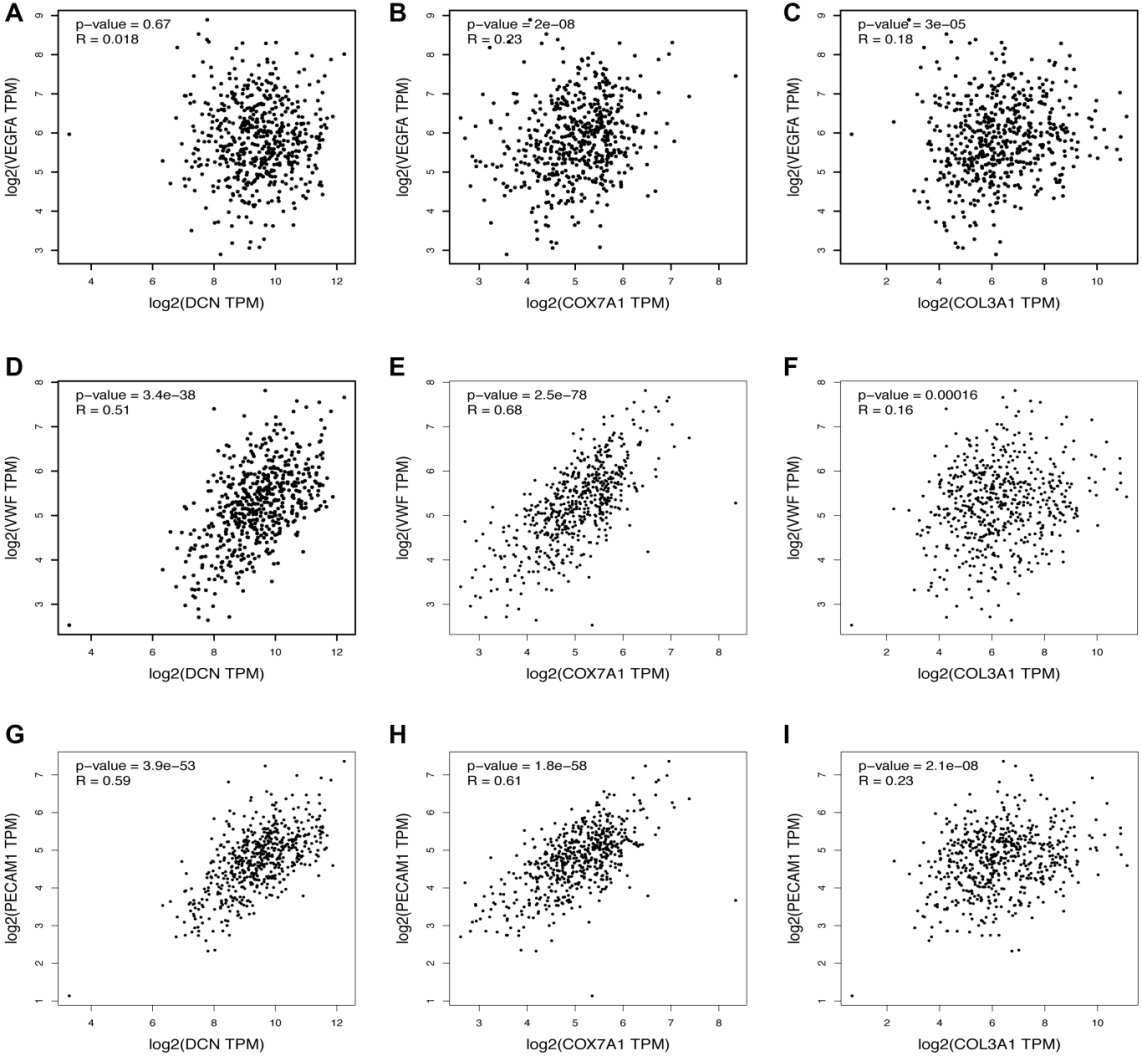
**Relationship between the upregulated DEGs and angiogenesis.** The correlation between the VEGFA and the expression of (**A**) DCN, (**B**) COX7A1, (**C**) COL3A1. The correlation between the VWF and the expression of (**D**) DCN, (**E**) COX7A1, (**F**) COL3A1. The correlation between the PECAM1 and the expression of (**G**) DCN, (**H**) COX7A1, (**I**) COL3A1.

**Figure 5:**
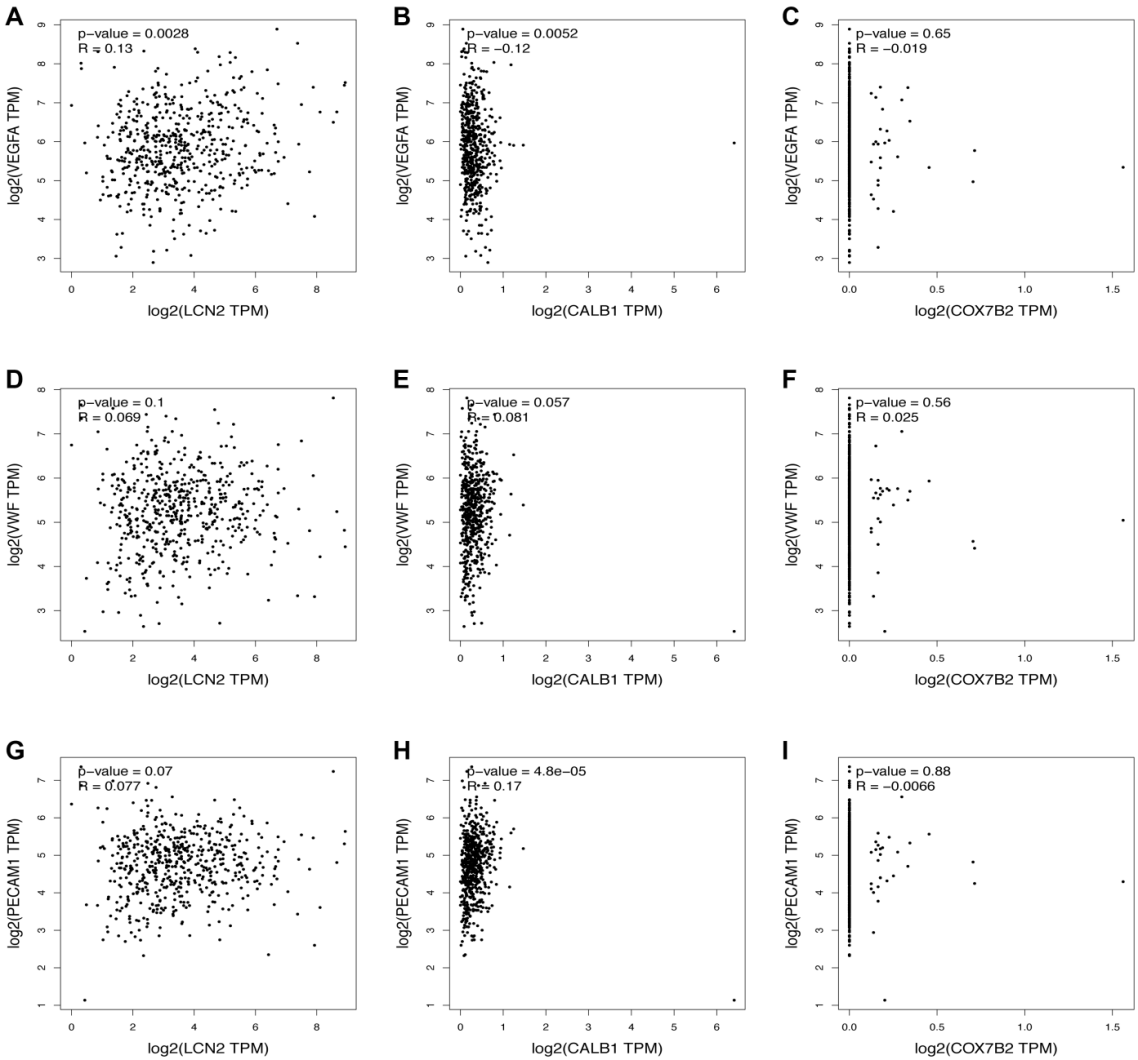
**Relationship between the downregulated DEGs and angiogenesis.** The correlation between the VEGFA and the expression of (**A**) LCN2, (**B**) CALB1, (**C**) COX7B2. The correlation between the VWF and the expression of (**D**) LCN2, (**E**) CALB1, (**F**) COX7B2. The correlation between the PECAM1 and the expression of (**G**) LCN2, (**H**) CALB1, (**I**) COX7B2.

The top three most upregulated or downregulated DEGs with protein-coding functions and the top five hub genes were selected, and the correlations between the DEGs and hub genes and the markers of angiogenesis, necroptosis, pyroptosis, ferroptosis, and anti-inflammatory activities in skin tissue were further analyzed using the GEPIA database. Notably, the vascularization correlation results of the upregulated DEGs and hub genes (Figure 4 and Supplementary Figure 1) suggested that almost all upregulated DEGs and hub genes were strongly positively correlated with vascularization markers (*P* < 0.001, R > 0). Regarding the downregulated DEGs (Figure 5), most were not significantly correlated with vascular markers. However, LNC2 was positively correlated with VEGFA expression (*P* ≤ 0.01, R = 0.13). CALB1was negatively correlated with VEGFA (*P* < 0.01, R = –0.12) and positively correlated with PECAM1 (*P* < 0.001, R = 0.17). Regarding the downregulated hub genes, almost all were positively correlated with VEGFA (*P* < 0.05, R > 0; Supplementary Figure 2A–2E). Interestingly, all downregulated hub genes were strongly negatively correlated with VWF and PECAM1 (*P* < 0.001, R < 0; Supplementary Figure 2F–2O).

For the correlations of the upregulated DEGs/hub genes and the markers of necroptosis, pyroptosis, ferroptosis, and anti-inflammatory activities (Table 3 and Supplementary Table 1), the results indicated that most of the genes were strongly positively correlated with the markers of necroptosis, ferroptosis, and anti-inflammatory activities (*P* < 0.05, R > 0), and DCN, COX7A1, and FNA1 were highly negatively correlated with pyroptosis markers (*P* < 0.01, R < 0), whereas COL3A1 and most other upregulated hub genes were positively correlated with the pyroptosis markers (*P* < 0.05, R > 0). For correlations of the downregulated DEGs/hub genes and the markers of necroptosis, pyroptosis, ferroptosis, and anti-inflammatory activities (Table 4 and Supplementary Table 2), the results showed that CALB1 and most of the hub genes were highly positively correlated with the necroptosis markers of caspase-8 (Casp8; *P* < 0.001, R > 0), whereas LCN2, CCNB1, and CDC20 were negatively correlated with the necroptosis markers of receptor-interacting protein kinase (RIPK1; *P* < 0.05, R < 0). LCN2, COX7B2, and all downregulated hub genes were positively correlated with pyroptosis markers (*P* < 0.05, R > 0), while CALB1 was negatively correlated (*P* < 0.01, R < 0). LCN2 and CALB1 were significantly positively correlated with the ferroptosis markers (*P* < 0.001, R > 0), while COX7B2 and all downregulated hub genes were not significant (*P* > 0.05). The downregulated hub genes of CCNB1, AURKB, and CDC20 were strongly negatively correlated with the anti-inflammatory markers of interleukin (IL)-10 (*P* < 0.01, R < 0), while all downregulated DEGs and other downregulated hub genes were not significantly correlated with the anti-inflammatory markers (*P* > 0.05).

**Table 3:** Correlations between upregulated DEGs and gene markers of necroptosis, pyroptosis, ferroptosis and anti-inflammatory in skin tissues.

**Mechanism**	**Gene marker**	**DCN**		**COX7A1**		**COL3A1**	
		**R**	***P***	**R**	***P***	**R**	***P***
**Necroptosis**	Casp8	0.25	^***^	–0.063	0.14	0.27	^***^
	RIPK1	0.24	^***^	–0.067	0.11	0.093	^*^
**Pyroptosis**	IL-1α	–0.2	^***^	–0.23	^***^	–0.031	0.46
	Casp1	–0.12	^**^	–0.21	^***^	0.28	^***^
**Ferroptosis**	GPX4	0.46	^***^	0.67	^***^	0.18	^***^
	NCOA4	0.32	^***^	–0.096	^*^	0.19	^***^
**Anti-inflammatory**	IL-4	0.12	^**^	0.05	0.24	0.15	^***^
	IL-10	0.36	^***^	0.32	^***^	0.11	^*^

**Table 4:** Correlations between downregulated DEGs and gene markers of necroptosis, pyroptosis, ferroptosis and anti-inflammatory in skin tissues.

**Mechanism**	**Gene marker**	**LCN2**		**CALB1**		**COX7B2**	
		**R**	***P***	**R**	***P***	**R**	***P***
**Necroptosis**	Casp8	0.068	0.11	0.18	^***^	0.0047	0.91
	RIPK1	-0.15	^***^	0.27	^***^	0.014	0.74
**Pyroptosis**	IL-1α	0.28	^***^	-0.13	^**^	0.092	^*^
	Casp1	0.13	^**^	-0.16	^***^	0.036	0.4
**Ferroptosis**	GPX4	0.15	^***^	-0.042	0.32	0.021	0.62
	NCOA4	-0.049	0.25	0.27	^***^	0.0036	0.93
**Anti-inflammatory**	IL-4	-0.038	0.37	0.034	0.42	0.074	0.079
	IL-10	0.047	0.26	-0.0096	0.82	0.0026	0.95

### Effects of seawater and hADSCs on human umbilical vein endothelial cells (HUVECs)

**Figure 6:**
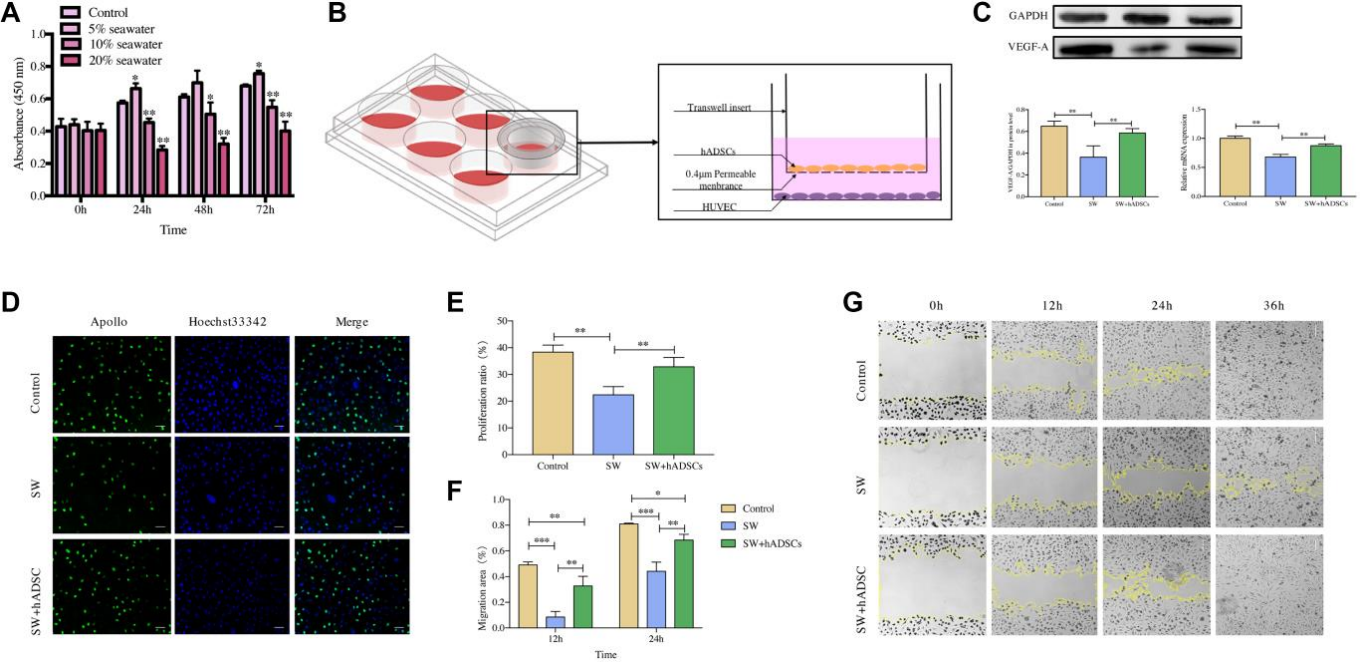
**Effect of hADSCs on the proliferation and migration of HUVEC *in vitro*.** (**A**) CCK8 assay results showed that the proliferation of HUVEC was significantly inhibited following exposure to 10% seawater concentration. (**B**) transwell coculture system schematic. (**C**) the effects of seawater and hADSC on the protein and mRNA expression of VEGFR in HUVEC. (**D–E**) the effects of seawater and hADSC on HUVEC proliferation. Proliferating HUVEC were stained with green fluorescence, and all nuclei were stained with blue fluorescence. Scale bars indicate 100 μm. (**F–G**) the effects of seawater and hADSC on HUVEC migration. ^*^P < 0:05, ^**^P < 0:01 and ^***^P < 0:001.

The proliferation and migration ability of vascular endothelial cells play a vital role in wound vascularization. Therefore, the effects of the coculture of seawater and hADSCs on the proliferation and migration of HUVECs were investigated. The results of the cell counting kit-8 (CCK8) assay (Figure 6A) showed that cell proliferation activity was significantly inhibited at 10% and 20% seawater concentrations (*P* < 0.01), and the inhibitory effect continued to exist as culture time increased. Therefore, 10% seawater concentration was used as the experimental concentration for subsequent experiments.

The expression of VEGFR is closely related to the vascularization of skin wounds thus, the effects of seawater and hADSCs on the protein and mRNA expression levels of VEGFR in HUVECs were detected (Figure 6C) [10, 11]. Expression levels of VEGFR were significantly lower in the SW group than those in the control group but were significantly higher in the SW + hADSC group (*P* < 0.01).

5-Ethynyl-2′-deoxyuridine (EdU) and cell scratch assays were used to evaluate the effects of seawater and hADSCs on HUVEC proliferation and migration, respectively. The EdU results (Figure 6D, 6E) indicated that the proliferation efficiency of HUVECs was significantly inhibited in the simulated seawater environment, while co-culture with hADSCs significantly improved the proliferation activity (*P* < 0.01). The cell scratch test results (Figure 6F, 6G) showed that compared with the control group, HUVEC scratches in the SW group had a significant delay in closure at 12 h, and co-culture with hADSCs relieved the effect of seawater on cell migration and significantly promoted cell migration ability (*P* < 0.01). Moreover, HUVEC scratches in the control and SW + hADSC groups were mainly healed at 36 h, while part of the unhealed area remained in the SW group.

## DISCUSSION

Marine workers are susceptible to various open injuries [12]. The wounds immersed in seawater that have large numbers of inflammatory factors, such as IL-8, TNF, and NO, in the injured area [13, 14]. The wounds continue to aggravate, and can eventually lead to mortality. In our previous study [9], a model of seawater immersion wounds was established, and it was observed that the healing efficiency of seawater immersion wounds compared with that of normal skin wounds was significantly delayed and produced refractory wounds. At the same time, hADSCs significantly accelerated the healing of seawater-immersed skin wounds by promoting skin stem cell proliferation and migration. However, the proangiogenic effects of hADSCs in seawater-immersed wounds have not, to our knowledge, previously been reported. Therefore, vascularization of skin wounds in each group was observed and compared. CD31, also known as PECAM1, is an important component of endothelial cell intercellular junctions and participates in angiogenesis. hADSCs differentiated into vascular endothelial cells and significantly promoted the vascularization of seawater-immersed wounds compared to those of the other two groups (*P* < 0.001). hADSCs can be differentiated into vascular endothelial cells for the treatment of ischemic vascular disease [15, 16]. Due to the blockage of vascularization in chronic wounds, there are insufficient neovascularization networks to provide nutrition and oxygen support for the cells in the wound healing process [17]. Thus, hADSCs and their derivatives may be involved in a new strategy for chronic wound treatment.

Angiogenesis can be defined as new blood vessel formation from preexisting vessels via sprouting or intussusception, which is a fundamental process in wound repair [18]. To explore the vascularization mechanism of hADSCs, GSE48228 microarray data were downloaded from the GEO database, and a total of 4,642 upregulated and 5,541 downregulated genes in hADSCs were identified by differential analysis compared with fibroblast controls. DCN was the main upregulated DEG, and LCN2 was the main downregulated DEG. Then, GO annotation and KEGG pathway enrichment analyses were performed to explore the biological functions of DEGs in skin wound healing. Notably, the upregulated DEGs were directly related to angiogenesis (GO:0001525), while the downregulated DEGs seemed to be involved in the repair process by regulating the cell cycle. Interestingly, the upregulated DEGs were mainly involved in the ECM remodeling process and the signal pathways of ECM-receptor interaction and PI3K-Akt. ECM components are multifunctional three-dimensional networks of interconnected macromolecules that provide support for the integrity of each stage of wound healing and act as a reservoir and regulator for the interaction of cytokines and growth factors [19, 20], which also play a crucial role in promoting blood vessel formation and maintaining blood vessel homeostasis [21]. The vascular ECM is composed of the basement membrane and interstitial ECM. The vascular basement membrane is largely comprised of laminin, collagen, perlecan, nidogen, and fibronectin, and provides the scaffolding for a pivotal process in vascular development and homeostasis. The vascular interstitial ECM is a reservoir for numerous growth factors, including VEGF, IGF, FGF and HGF, and is necessary for sprouting angiogenesis and vascular development [22]. Activation of the PI3K-Akt signaling pathway can promote the formation of blood vessels by upregulating the expression of VEGF [23–25]. For the downregulated DEGs, downregulation of cell cycle-related genes can significantly inhibit angiogenesis in tumor tissue, but its effect on the vascularization of skin wounds has not yet, to our knowledge, been fully elucidated.

Hub genes may play an important role in the onset and progression of wound healing. Through analysis of the PPI network of DEGs, several hub genes with high node degrees were identified. Then, the top five hub genes with the highest node degree and the top three DEGs with protein-coding functions were selected, and the correlations of hub genes/DEGs and the markers of angiogenesis, necroptosis, pyroptosis, ferroptosis, and anti-inflammatory activities in skin tissue were analyzed. Programmed cell death, including necroptosis, pyroptosis and ferroptosis, acts as an important role in host defense and tissue homeostasis as well as in the occurrence and development of various diseases [26–28]. Casp8 was identified as a crucial mechanism for relieving inflammation in epithelial barriers, and RIPK1 could be regarded as a scaffold to counteract the activation of necroptosis, further maintaining tissue integrity and inhibiting inflammation in the skin [29]. In this study, this may have been due to the low specificity of Casp8, which was strongly positively correlated with most upregulated and downregulated genes. However, PIPK1 was strongly positively correlated with most upregulated genes and negatively correlated with downregulated genes. Pyroptosis plays a critical role in the development of autoinflammatory conditions and cancer [30], and excessive inflammation of skin wounds can delay wound healing and form pathological keloids [31]. The inflammasome is the central link that causes pyroptosis thus, Casp1 and IL-1α, the key components of the inflammasome, were selected in this study. We found that the most upregulated DEGs and all downregulated hub genes were negatively and positively correlated with pyroptosis markers, respectively. Ferroptosis is a newly-identified form of regulated cell death, defined as the iron-dependent accumulation of lipid reactive oxygen species [32], that can significantly promote the formation of tumor blood vessels [33]. GPX4 uses glutathione to protect cells from ferroptosis by removing phospholipid peroxide [34], while NCOA4 mediates ferritin degradation to support ferroptosis [35]. For the upregulated DEGs and hub genes, most were positively correlated with ferroptosis markers. However, all downregulated hub genes were strongly positively correlated with NCOA4 and strongly negatively correlated with GPX4. These results implied that the downregulated hub genes may play major roles in regulating programmed cell death. The production of IL-4 is the primary pathway for the development and maintenance of wound-healing macrophages, which secrete components of the ECM and is thus, closely related to wound healing [36]. IL-10 inhibits the production and activity of various proinflammatory cytokines and promotes the healing of skin wounds by regulating the local inflammation response [37]. Our results indicated that the upregulated DEGs/hub genes were positively correlated with IL-4 and IL-10, while downregulated hub genes were mainly negatively correlated with IL-10, which suggests that hADSCs inhibit wound inflammation.

The proliferation and migration of vascular endothelial cells were positively correlated with the degree of vascularization during wound repair. Our results indicated that the upregulated DEGs/hub genes were positively correlated with vascularization markers, while downregulated hub genes were negatively correlated with vascularization markers. These results are consistent with those of our *in vitro* experiments. We co-cultured hADSCs with HUVECs and found that hADSCs significantly promoted the proliferation and migration ability of HUVECs and VEGFA mRNA expression. Our results provide a basis for the mechanism by which hADSCs promote wound vascularization. However, in the co-culture system, ADSCs do not directly contact the vascular endothelial cells. Therefore, ADSCs are more likely to promote the biological functions of vascular endothelial cells by secreting growth factors or exosomes. ADSCs can paracrine a variety of angiogenesis-related cytokines, such as bFGF, VEGF, HGF, and PDGF, to promote local microvascularization [8, 38]. Unfortunately, growth factor therapy is not currently considered an effective treatment based on preclinical and clinical studies [39]. Exosomes types of membranous vesicles with a diameter of approximately 30–100 nm that are released from cells into the ECM [40]. Stem cell exosomes can carry a variety of growth factors and noncoding RNAs, which serve as a medium for cell-to-cell communication and target the biological functions of cells [41, 42]. Therefore, we hypothesized that in co-culture systems, ADSCs secrete exosomes rich in numerous DEGs and noncoding RNAs that inhibit programmed cell death to promote the proliferation and migration of vascular endothelial cells. Additionally, further *in vitro* or *in vivo* studies should be performed to validate our results.

The present study has several limitations. The appropriate inflammatory response in the early stages of skin wounds can resist the damage caused by pathogenic microorganisms and promote wound healing. However, excessive or prolonged inflammatory responses lead to delayed wound healing and scar formation. Therefore, the specific mechanism of hADSC regulation of wound inflammation needs to be further explored.

In summary, we found that hADSCs can differentiate into vascular endothelial cells and promote seawater-immersed wound vascularization. Then, we analyzed the DEG expressions between hADSCs and fibroblast controls using bioinformatics. These DEGs were found to potentially promote wound healing by regulating the mechanisms of ECM remodeling, programmed cell death, inflammation, and vascularization. Our results provide novel insights into the use of hADSCs in the regeneration of seawater-immersed skin wounds and chronic wounds.

## MATERIALS AND METHODS

### Immunofluorescence observations

To observe the differentiation effects of hADSCs, the cells used for injection therapy were labeled with red fluorescence with CM-DiL fluorescent dye (Invitrogen, Thermo Scientific, USA) in advance. On the day 14 after injection treatment, wounded skin tissues of the SW + hADSCs group were immediately excised and frozen sections of 10 μM were prepared. Frozen sections were dried for 15 min at room temperature, then soaked for 10 min in PBS solution to remove tissue glue and incubated with CD31 antibody (10 μg/mL, R&D Systems, USA) at 4°C overnight. FITC-labeled goat anti-mouse IgG (H + L) cross-adsorbed secondary antibody (Beyotime Biotechnology Company, Jiangsu, China) and 1X Hoechst33342 (Ribo, Guangzhou, China) were incubated with the slices for 4 h and 30 min, respectively. The slices were then washed with PBS and mounted with antifade mountant (ProLong, Thermo Scientific, USA). Images were acquired using a Zeiss fluorescence microscope (HLA100, Shanghai, China).

### Immunohistochemistry observations

To observe the vascularization effect of hADSCs in repairing seawater-immersed wounds, wound skin tissues on days 14 and 21 were used to prepare stained sections. The wound tissues were initially made into paraffin sections through formaldehyde treatment, dehydration, paraffin embedding, and sectioning. Paraffin sections were dewaxed and antigens were repaired, blocked, and incubated with CD31 primary antibody (10 μg/mL, R&D Systems, USA) at 4°C overnight. Then, the sections were incubated for 4 h with secondary antibodies (Cell Signaling Technology, MA, USA) and washed with PBS. The sections were incubated with DAB chromogen and counterstained with hematoxylin for immunohistochemical analysis. Sections were photographed using an optical microscope (Olympus, Japan) and quantitative analysis was performed using Image-Pro Plus software (Medical Cybernetics, USA).

### Cell proliferation assay

HUVECs were obtained from the American Type Culture Collection (Manassas, VA, USA) and cultured in high-glucose Dulbecco’s modified Eagle’s medium (HyClone, Utah, USA) mixed with 10% fetal bovine serum (Gibco, Grand Island, USA), and the medium was changed every two-to-three days.

To explore the effect of the co-culture of seawater and hADSCs on cell proliferation. HUVECs were seeded into 96-well plates at a density of 2000 cells/well. After the cells were cultured for 12 h, the medium was replaced with a high-glucose culture medium containing different proportions of seawater (volume fractions of 5%, 10%, and 20%) for 24, 48, or 72 h. Artificial seawater was configured as previously reported. At each observation time point, 10 μL of CCK8 reagent (Beyotime Biotechnology Company, Shanghai, China) was added to each well and incubated for 2 h. At the end of incubation, absorbance was measured at 450 nm using an enzyme-labeled instrument (Tecan, Shanghai, China).

First, 0.4-μM Transwell polycarbonate membranes (Corning, New York, USA) were used to realize the co-culture of hADSCs and HUVECs (Figure 6B), and the proliferation rate of HUVECs was detected using an EdU kit (Ribo, Guangzhou, China). HUVECs were seeded into 24-well plates at 2 × 10^5^ cells/well. When confluency reached 70–80%, the cells were divided into three groups; control, SW, and SW + hADSC. Cells of the control group were cultured in complete medium, and cells of the SW and SW + hADSC groups were cultured in complete medium mixed with seawater. After 48 h of incubation, the cell proliferation ratio of each group was determined using the EdU kit. Fluorescence staining was performed according to the manufacturer's protocol. The results were photographed with a Zeiss fluorescence microscope and analyzed using ImageJ software (Bethesda, MD, USA).

### Cell migration assay

A cell scratch assay was used to explore the effect of the co-culture of seawater and hADSCs on cell migration. HUVECs were inoculated in six-well plates with 1 × 10^6^ cells/well and cultured to 80–90% confluency. The groupings and treatments were the same as those used in the EdU assay. The cell monolayer was scratched with a 10 μL sterile pipette tip quickly and washed two-to-three times with PBS. At 0, 12, 24, and 36 h observation time points, the scratch area was photographed using a Zeiss inverted phase contrast microscope (Observer D1, Shanghai, China) and measured using ImageJ software. The following formula was used to assess cell migration ability:

$$\text{Migration area (\%)}=\frac{\text{S}0-\text{Sx}}{\text{S}0}\times 100\%$$

where S0 represents the initial scratch area (*t* = 0 h) and Sx represents the residual scratch area at the time of measurement (*t* = n h).

### Quantitative real-time polymerase chain reaction (qRT-PCR)

HUVEC groupings and treatments were the same as those in the EdU assay. HUVECs were co-cultured with seawater and hADSCs for 72 h, then total RNA from each group was extracted with Trizol reagent (Vazyme, Jaingsu, China). Reverse transcription of 2 μg of total RNA into cDNA was used as a template for qRT-PCR. Using GAPDH as an internal reference gene, the normalized relative expression level of VEGFR was calculated using the 2^-ΔΔCt^ method. GAPDH forward primer sequence: 5'-TGTGGGCATCAATGGATTTGG-3'; reverse primer sequence: 5'-ACACCATGTATTCCGGGTCAAT-3'. VEGFR forward primer sequence: 5'-AGCAAAGGGTGGAGGTG-3'; reverse primer sequence: 5'-ACATAAATGACCGAGGC-3'. The reaction conditions were 95°C pre-denaturation for 30 s followed by 40 cycles of 95°C for 10 s, 60°C for 10 s, and 72°C for 10 s.

### Western blotting

The proteins for western blotting from each sample were extracted and separated by PAGD and transferred to a PVDF membrane (Cell Signaling Technology, MA, USA) at 300 mA for 2 h. The membrane was initially incubated in blocking buffer (5% bovine serum albumin) for 2 h at room temperature then incubated overnight at 4°C with primary GAPDH (1:1000, Cell Signaling Technology, MA, USA) or VEGF-A antibody (1:1000, Abcam, Cambridge, MA, UK) followed by 2 h of incubation with the appropriate horseradish peroxidase-conjugated secondary antibodies (1:3000, Beyotime, China). Protein expression was visualized by chemiluminescence using an Alpha Imager scanner (Tecan, Thermo Fisher Scientific, USA).

### Differential analysis of gene expression in hADSCs

The gene expression microarray dataset (GSE48228), comprised of samples from six hADSCs, one fibroblast, and one endothelial cell, was downloaded from the Gene Expression Omnibus (GEO) database (https://www.ncbi.nlm.nih.gov/geo). The dataset was based on the GPL6480 platform (Agilent-014850 Whole Human Genome Microarray), and the mRNA expression data of hADSCs and fibroblast control were downloaded and used for this study. The platform data were converted using R language software and standardized using the limma R package’s array function (http://www.bioconductor.org/). The differential analysis of mRNAs in hADSCs compared with fibroblasts was performed using the GEO2R analysis tool. An mRNA with a *p* value of < 0.05 and a base-2 log FC value greater than ± 2 was defined as a DEG, and the integrated DEG lists of upregulated and downregulated genes were saved for subsequent analysis.

### Integration analysis of the DEGs

The top 2000 most upregulated and downregulated DEGs were selected, and the PPI networks were analyzed using the STRING database (https://string-db.org/) and visualized using Cytoscape (version 3.7.2) software [43]. Then, the degree analysis method in the CytoHubba plug-in was used to calculate the grade nodes in the PPI network, in which highly connected nodes were defined as hub genes, and the top 20 hub genes were selected and visualized using Cytoscape.

The DAVID (https://david.ncifcrf.gov/home.jsp) provides a comprehensive set of functional annotation tools to understand the biological meaning behind a large list of genes [44]. In this study, the DAVID was used for GO annotation and KEGG pathway enrichment analysis of the integrated DEGs. GO annotation analysis of DEGs involved the BP, CC, and MF.

### Correlations between DEGs and gene markers of angiogenesis, necroptosis, pyroptosis, ferroptosis, and anti-inflammatory activities in GEPIA

The relationship between DEGs and gene markers of proangiogenic activity, necroptosis, pyroptosis, ferroptosis, and anti-inflammatory activity was determined using the GEPIA database (http://gepia.cancer-pku.cn/index.html) [45]. The gene markers, including VEGFA, VWF, PECAM1, Casp8, RIPK1, IL-1α, Casp1, Glutathione peroxidase-4 (GPX4), nuclear receptor coactivator-4 (NCOA4), IL-4, and IL-10, were selected from previous studies [35, 36, 46, 47]. The GEPIA dataset, an interactive online platform with normal sample information from the Genotype Tissue Expression project, was used for the correlation analysis of the DEGs and gene markers in normal skin tissues. The gene expression level was adjusted with log2 TPM, and Spearman’s method was used to determine the correlation coefficient. DEGs are plotted on the x-axis, while the gene markers of interest are plotted on the y-axis.

### Statistical analysis

Data were analyzed with SPSS 17.0 and are presented as means ± standard deviation. Significance was determined by ANOVA, and the correlation analysis of gene expression was evaluated using Spearman’s rank test. A *p* value of < 0.05 was considered significant.

## Supplementary Material

Supplementary Figures

Supplementary Tables
