# Supplementary material for: Human adipose-derived stem cells promote seawater-immersed wound healing via proangiogenic effects
Source: Aging (Albany NY). 2021 Mar 26;13(13):17118–36. doi: 10.18632/aging.202773 (PMC8312430; doi:10.18632/aging.202773)
Supplement: Supplementary Figures [file aging-13-202773-s001.pdf]

SUPPLEMENTARY FIGURES

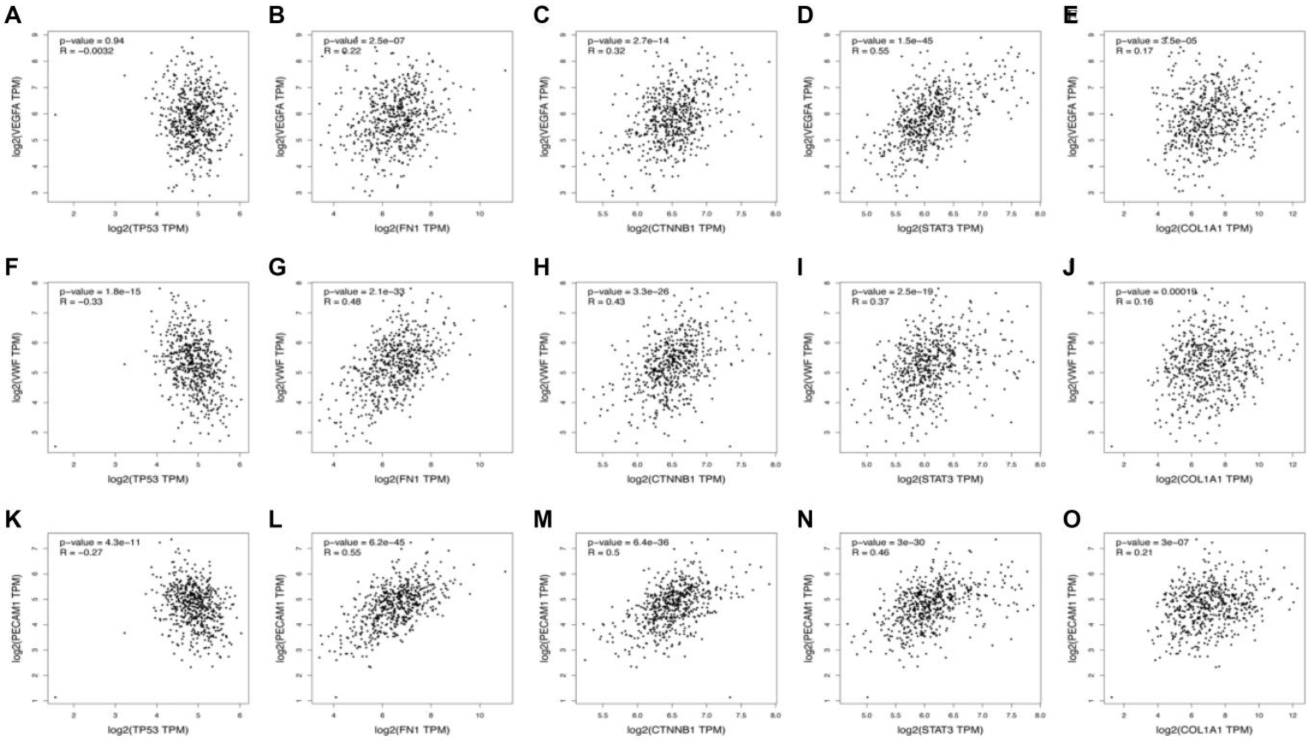

Supplementary Figure 1. Relationship between the upregulated hub genes and angiogenesis. The correlation between the VEGFA and the expression of (**A**) TP53, (**B**) FN1, (**C**) CTNNB1, (**D**) STAT3, (**E**) COL1A1. The correlation between the VWF and the expression of (**F**) TP53, (**G**) FN1, (**H**) CTNNB1, (**I**) STAT3, (**J**) COL1A1. The correlation between the PECAM1 and the expression of (**K**) TP53, (**L**) FN1, (**M**) CTNNB1, (**N**) STAT3, (**O**) COL1A1.

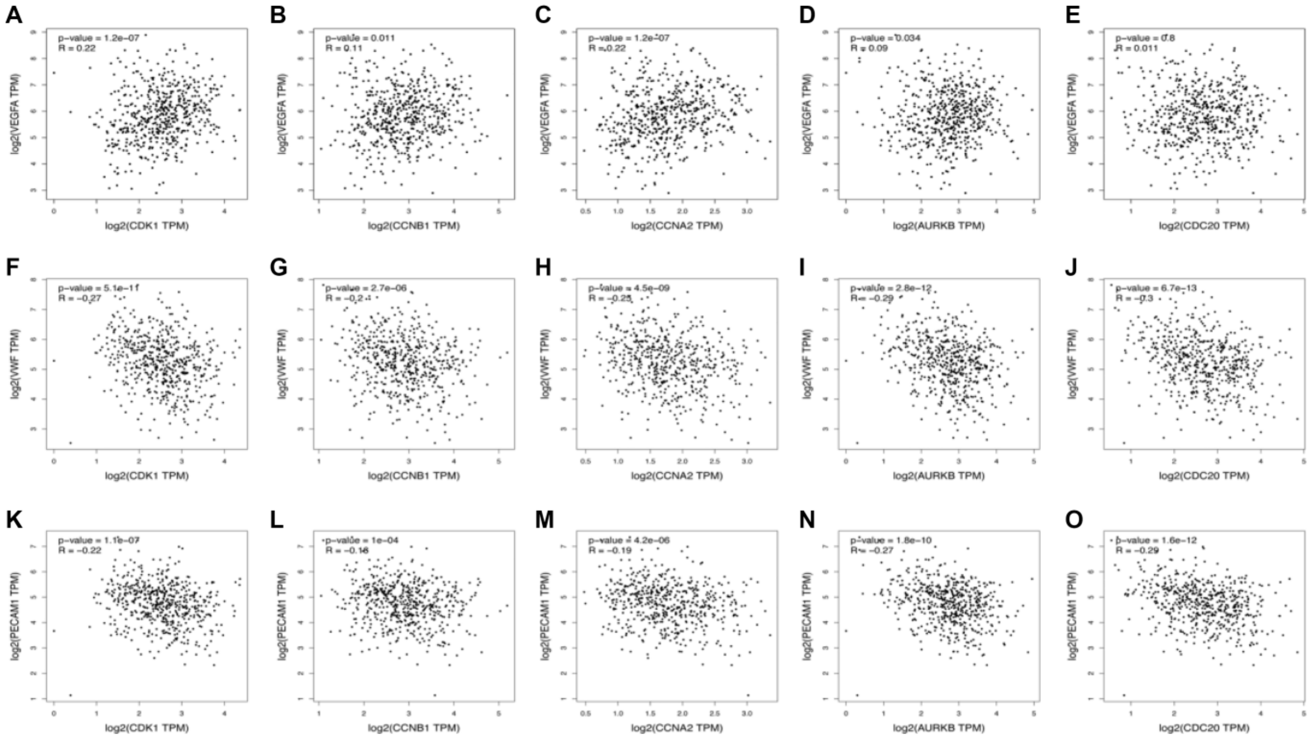

Supplementary Figure 2. Relationship between the downregulated hub genes and angiogenesis. The correlation between the VEGFA and the expression of (**A**) CDK1, (**B**) CCNB1, (**C**) CCNA2, (**D**) AURKB, (**E**) CDC20. The correlation between the VWF and the expression of (**F**) CDK1, (**G**) CCNB1, (**H**) CCNA2, (**I**) AURKB, (**J**) CDC20. The correlation between the PECAM1 and the expression of (**K**) CDK1, (**L**) CCNB1, (**M**) CCNA2, (**N**) AURKB, (**O**) CDC20.
