# Supplementary material for: Human adipose-derived stem cells promote seawater-immersed wound healing via proangiogenic effects
Source: Aging (Albany NY). 2021 Mar 26;13(13):17118–36. doi: 10.18632/aging.202773 (PMC8312430; doi:10.18632/aging.202773)
Supplement: Supplementary Tables [file aging-13-202773-s002.pdf]

SUPPLEMENTARY TABLES

Supplementary Table 1. Correlations between hub genes of up-regulated genes and gene markers of necroptosis, pyroptosis, ferroptosis and anti-inflammatory in skin tissues in GEPIA.

Mechanism	Gene marker	TP53		FN1		CTNNB1		STAT3		COL1A1	
		R	*P*	R	*P*	R	P	R	*P*	R	*P*
Necroptosis	Casp8	–0.017	0.7	0.21	***	0.17	***	0.25	***	0.28	***
	RIPK1	0.12	**	0.055	0.2	0.12	**	0.044	0.3	0.086	*
Pyroptosis	IL-1α	0.14	***	–0.12	**	0.031	0.46	0.14	***	–0.055	0.2
	Casp1	0.47	***	0.014	0.75	0.24	***	0.14	*	0.23	***
Ferroptosis	GPX4	–0.31	***	0.44	***	0.31	***	0.28	***	0.2	***
	NCOA4	0.33	***	0.14	***	0.32	***	0.086	*	0.11	*
Anti-inflammatory	IL-4	0.047	0.27	0.11	*	0.12	**	–0.067	0.12	0.12	**
	IL-10	–0.29	***	0.22	***	0.22	***	0.26	***	0.12	**

Supplementary Table 2. Correlations between hub genes of down-regulated genes and gene markers of necroptosis, pyroptosis, ferroptosis and anti-inflammatory in skin tissues in GEPIA.

Mechanism	Gene marker	CDK1		CCNB1		CCNA2		AURKB		CDC20	
		R	*P*	R	*P*	R	*P*	R	*P*	R	*P*
Necroptosis	Casp8	0.28	***	0.15	***	0.2	***	0.2	***	0.056	0.19
	RIPK1	–0.024	0.57	–0.099	*	–0.082	0.054	–0.045	0.29	–0.13	**
Pyroptosis	IL-1α	0.34	***	0.28	***	0.31	***	0.21	***	0.19	***
	Casp1	0.51	***	0.14	***	0.5	***	0.35	***	0.35	***
Ferroptosis	GPX4	–0.24	***	–0.18	***	–0.16	***	–0.24	***	–0.21	***
	NCOA4	0.23	***	0.19	***	0.2	***	0.12	**	0.12	**
Anti-inflammatory	IL-4	0.026	0.55	0.056	0.19	0.067	0.11	0.022	0.61	0.017	0.7
	IL-10	–0.07	0.099	–0.12	**	–0.065	0.12	–0.19	***	–0.21	***
